# FOXP2 suppresses gastric cancer progression by transcriptionally repressing FBXW2 via WASL degradation

**DOI:** 10.1038/s41420-025-02643-1

**Published:** 2025-07-28

**Authors:** Sihan Lin, Wencheng Kong, Xinchun Liu, Guang Yin, Kangwen Cheng, Zonglei Mao, Yuqiang Shan, Xinger Lv

**Affiliations:** 1 Department of Emergency Surgery, Zhejiang Provincial People’s Hospital, Hangzhou, Zhejiang PR China; 2 Department of Gastroenterological Surgery, Affiliated Hangzhou First People’s Hospital, School of Medicine, Westlake University, Hangzhou, Zhejiang PR China

**Keywords:** Protein-protein interaction networks, Oncogenesis

## Abstract

Gastric cancer (GC) is an aggressive malignancy with poor clinical outcome. F-box and WD repeat domain-containing protein 2 (FBXW2), a substrate receptor of the SKP1-Cullin 1-F-box (SCF) E3 ubiquitin ligase complex, has been implicated in tumor suppression across multiple malignancies; however, its role in GC progression remains undefined. Here, we integrated transcriptomic analyses using the TNMplot database and clinical specimens to demonstrate that FBXW2 expression was significantly downregulated in GC tissues, with low FBXW2 levels correlating closely with poor survival in GC patients. Functional characterization via gain- and loss-of-function strategies revealed that FBXW2 overexpression potently inhibited proliferation, cancer stem cell phenotype, migratory capacity, and invasive potential in human GC cell lines. Consistently, xenograft tumor models showed that FBXW2 overexpression delayed tumor growth and suppresses pulmonary metastasis. FBXW2 silencing promoted malignant progression both in vitro and in vivo. Label-free quantitative proteomics combined with mechanistic investigations identified WASP-like actin nucleation-promoting factor (WASL), a key regulator of cytoskeletal dynamics, as a direct downstream target of FBXW2. FBXW2 physically interacted with WASL and facilitated its ubiquitination-dependent proteasomal degradation. Ectopic WASL expression abrogated FBXW2-mediated suppression of GC cell viability and metastatic potential. Chromatin immunoprecipitation-PCR and DNA Pull Down analyses further revealed that Forkhead box P2 (FOXP2), a transcription factor frequently upregulated in GC, directly bound the FBXW2 promoter to repress its transcription, linking epigenetic dysregulation to FBXW2 downregulation in malignant tissues. Collectively, this study establishes FBXW2 as a critical tumor suppressor in GC, operating through ubiquitin-mediated degradation of WASL to inhibit cancer progression. Targeting the FOXP2-FBXW2-WASL axis may represent a promising therapeutic strategy for combating GC malignancy.

## Introduction

Gastric cancer (GC) ranks among the top prevalent malignancies globally with substantial morbidity and mortality, especially in China [1]. Gastric adenocarcinoma makes up about 95% of entire spectrum of GC cases [2]. GC exhibits marked tumor heterogeneity, contributing to metastasis, recurrence and resistance to chemoradiotherapy. Despite advances in endoscopic, surgical and comprehensive therapeutic regimens, overall five-year survival outcomes for GC patients remain unsatisfactory [2]. These clinical challenges underscore the pressing requirement for innovative therapeutic strategies to reduce the global burden of this disease.

F-Box and WD Repeat Domain Containing 2 (FBXW2; also called FBW2 or MD6), one of approximately 70 F-box proteins, functions as substrate adaptors within ubiquitin ligase complexes that are made up of S-Phase Kinase Associated Protein (SKP) 1, Cullin 1, and F-box proteins, thereby enabling the degradation of protein [3]. Structurally, FBXW2 harbors two functional domains : (1) an amino-terminal F-box domain that interacts with binding the linker protein SKP1, and (2) a carboxyl-terminal region harboring seven WD40 repeats responsible for substrate recognition and ubiquitination [4]. Emerging evidence indicates that FBXW2 acts as a tumor suppressor in multiple malignancies. For instance, FBXW2 induced ubiquitin-mediated proteolysis of β-catenin to delay invasive and migratory activities of lung cancer cells [5]. It also promoted ubiquitination and breakdown targeting SKP2, thereby repressing proliferation and in vivo tumorigenicity in lung cancer [6]. In prostate cancer, FBXW2 reduced epidermal growth factor receptor (EGFR) protein stability, thus suppressing cell proliferation and metastatic ability [7]. In breast cancer, FBXW2 inhibited tumor growth, stemness, and paclitaxel resistance by targeting NF-κB p65 degradation [8]. Paradoxically, Yin et al. reported that FBXW2 promoted tumor sphere formation and tamoxifen resistance in breast cancer cells through promoting muscle segment homeobox 2 (MSX2) ubiquitination [9]. Up till now, the functional significance of FBXW2 in GC remains unclear.

Neural Wiskott-Aldrich syndrome protein (N-WASP), otherwise called WASL, functions as an actin nucleation-promoting protein. WASL interacts with components of cellular cytoskeletal network, such as the actin-related protein 2/3 complex, to induce cytoskeletal filament assembly, consequently facilitating invasive behavior of tumor cells and membrane protrusion formation with matrix-degrading activity [10]. WASL exerts a crucial role in cancer metastasis. High WASL expression has been linked to the advancement of tumors and an unfavorable prognosis in patients with malignancies including lung, esophageal, liver, breast, and pancreatic malignancies [11–15]. Whether WASL-mediated tumor cell motility and metastatic dissemination affect the progression of GC is unknown.

Chromatin immunoprecipitation (ChIP) followed by microarray analysis performed by Elizabeth Spiteri et al. identified FBXW2 as a potential target of Forkhead Box P2 (FOXP2) transcriptional regulation [16]. As a member of Forkhead box transcription factor family, FOXP2 bound to the E-cadherin promoter and activated its transcription, thereby inhibiting breast cancer cell metastasis [17]. Accumulating evidence from prior investigations has demonstrated that FOXP2 is frequently downregulated in diverse tumors, where it exerts tumor-suppressive effects, such as in GC [18, 19]. These findings suggest that FBXW2 loss in GC may be mechanistically linked to FOXP2 downregulation.

The primary objective of this investigation was to elucidate the functional significance and molecular mechanisms of FBXW2 in GC. Through comprehensive functional genetic manipulation approaches in both in vitro (human GC cell lines) and in vivo (cell-derived tumor xenograft mouse models) systems, we systematically investigated the tumor-suppressive effects of FBXW2. Specifically, our study focused on two key aspects: (1) the potential involvement of WASL in mediating the anti-carcinogenic effects of FBXW2, and (2) the potential regulatory role of FOXP2 in FBXW2 downregulation in GC progression.

## Results

### FBXW2 is a downregulated gene associated with poor prognosis in GC

**Fig. 1:**
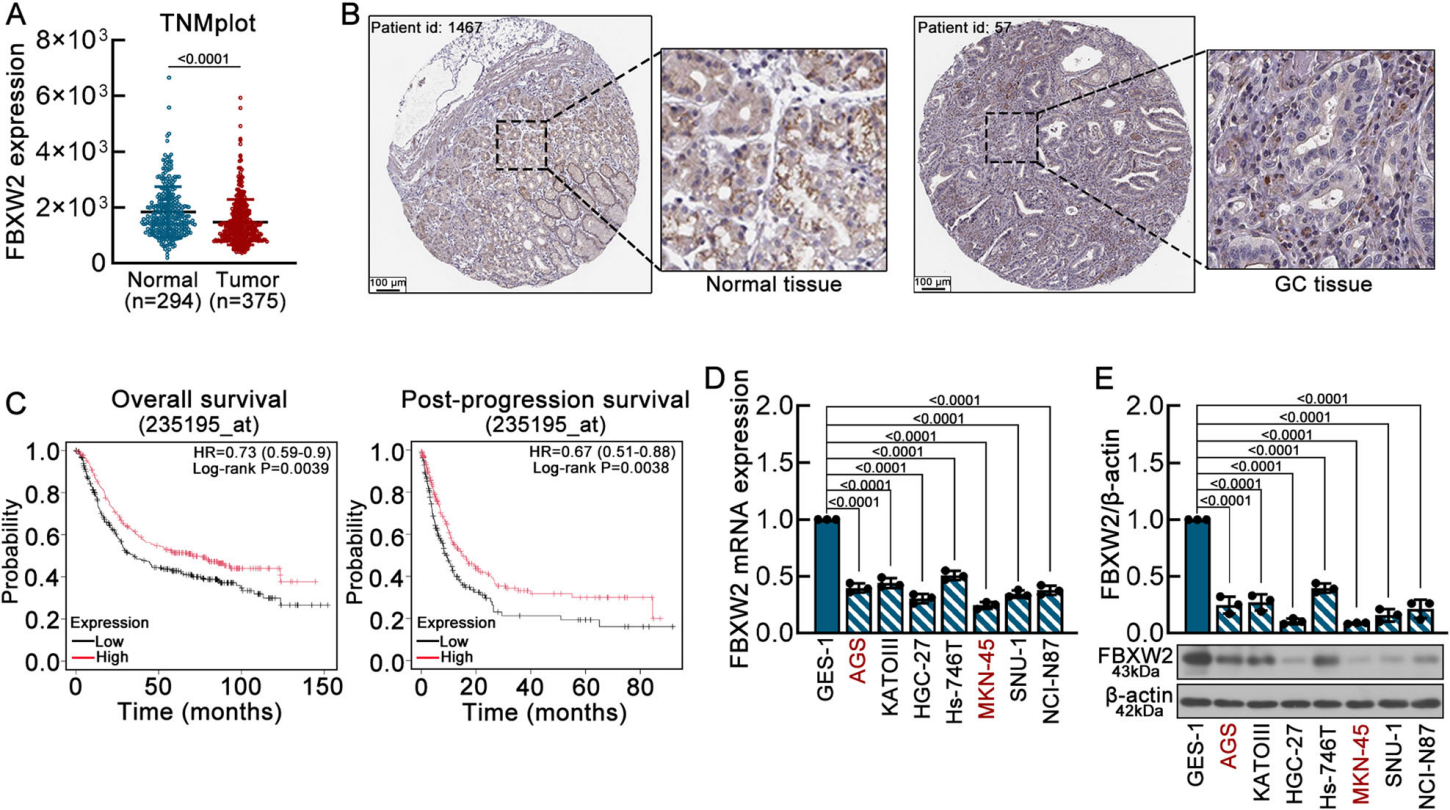
FBXW2 is a downregulated gene associated with poor prognosis in GC. **A** Expression of FBXW2 in the GC and normal tissues obtained from the TNMplot database. **B** Representative images of FBXW2 immunohistochemistry in normal and GC tissues obtained from the Human Protein Atlas database. Scale bars = 100 μm. **C** Overall survival and post-progression survival analysis of FBXW2 in GC patients obtained from the KM-plot database. **D** Relative mRNA expression levels of FBXW2 in the human gastric epithelial cell line GES-1 and seven GC cell lines (AGS, KATOIII, HGC-27, Hs-746T, MKN-45, SNU-1, NCI-N87) were determined by real-time PCR. **E** Western blotting analysis. Top, relative optical density of FBXW2 in the human gastric epithelial cell line GES-1 and seven GC cell lines (AGS, KATOIII, HGC-27, Hs-746T, MKN-45, SNU-1, NCI-N87) was quantified via Gel-Pro-Analyzer software; β-actin was used as the internal control. Bottom, representative protein bands. Data were expressed as mean ± SD.

RNA-seq data from the TNMplot database revealed significantly lower FBXW2 expression in gastric adenocarcinoma tissues compared to normal tissues (Fig. 1A). Immunohistochemical results using the HPA database confirmed reduced FBXW2 protein levels in gastric adenocarcinoma tissues (Fig. 1B). KM-plot displayed that GC patients with low FBXW2 expression had a worse prognosis (Fig. 1C). Therefore, FBXW2 might exert a crucial role in the carcinogenesis of GC. Basal FBXW2 expression was quantified in GC cell lines (AGS, KATOIII, HGC-27, Hs-746T, MKN-45, SNU-1, and NCI-N87) and normal gastric epithelial cells (GES-1). Real-time PCR and western blotting analyses revealed that the levels of FBXW2 mRNA (Fig. 1D) and protein (Fig. 1E) in all cancer cell lines were significantly lower than those in GES-1 cells. The MKN-45 and AGS cell lines were chosen for subsequent functional investigation due to their tumorigenicity and metastasis [20, 21].

### Overexpression of FBXW2 inhibits proliferation and in vivo tumorigenicity of GC cells

**Fig. 2:**
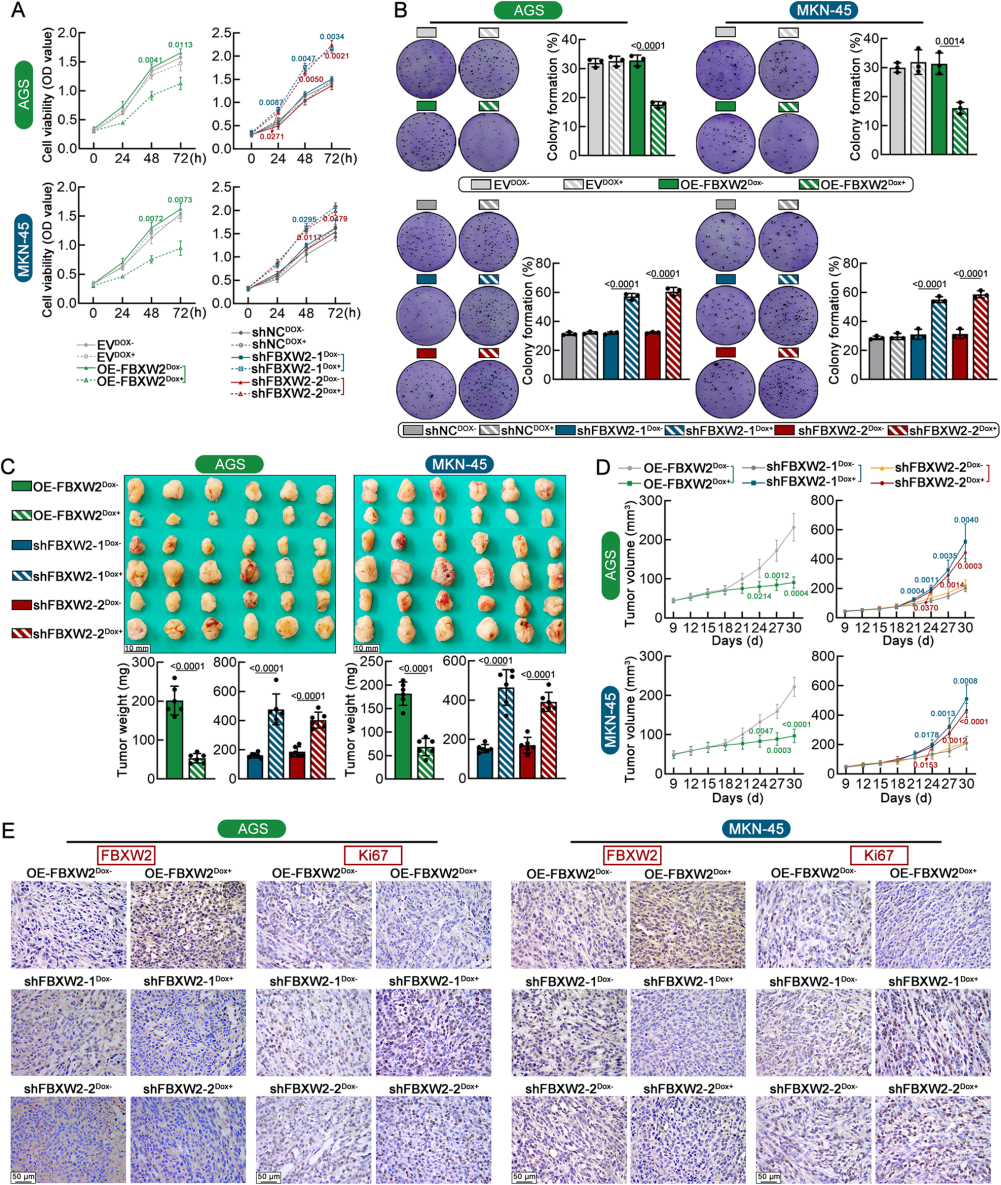
Overexpression of FBXW2 inhibits proliferation and in vivo tumorigenicity of GC cells. Lentivirus-infected GC cells were stably selected with puromycin and then treated with or without 2.5 μg/ml doxycycline (Dox). **A** The proliferation of GC cells with stable FBXW2 knockdown or overexpression was assessed using CCK-8 assays. **B** Colony formation capacity of GC cells was determined using plate colony formation assays. GC cells were injected subcutaneously into nude mice. After tumor establishment, mice were feed 2 mg/ml DOX in drinking water to induce FBXW2 expression. **C** Representative xenograft tumor images and tumor weight. Scale bars = 10 mm. **D** Starting on day 9 after injection, the tumor volume was determined every 3 days and the tumor growth curve was drawn. **E** Representative images of immunohistochemistry for FBXW2 and Ki67 in the tumor tissues (Scale bars = 50 μm, magnification: ×400). Data were expressed as mean ± SD.

Expression of FBXW2 in GC cells (AGS and MKN-45) was effectively regulated by lentiviral inducible expressing or shRNA vector in the presence of Dox (Supplementary Fig. S1). CCK-8 assay at the dictated time and colony formation assay revealed that FBXW2 manipulation (knockdown or overexpression) did not affect cell proliferation in the absence of Dox (Fig. 2A, B). Under Dox induction, high FBXW2 expression inhibited proliferation of both AGS and MKN-45 cells, while its low expression promoted cell proliferation (Fig. 2A, B).

Next, xenograft tumor formation in nude mice was analyzed to determine the contribution of FBXW2 to GC cell-derived tumorigenesis. With Dox induction, FBXW2 overexpression led to slower tumor growth and smaller tumor volume in comparison to that without Dox (Fig. 2C, D). Immunohistochemical analysis showed that FBXW2 overexpression enhanced FBXW2 immunoreactivity while suppressing the expression of cell proliferation marker Ki67 in tumor tissues (Fig. 2E). FBXW2 silencing yielded opposite alterations in vivo upon Dox induction (Fig. 2E).

### High FBXW2 expression curbs invasion, stemness and pulmonary metastasis of GC cells

**Fig. 3:**
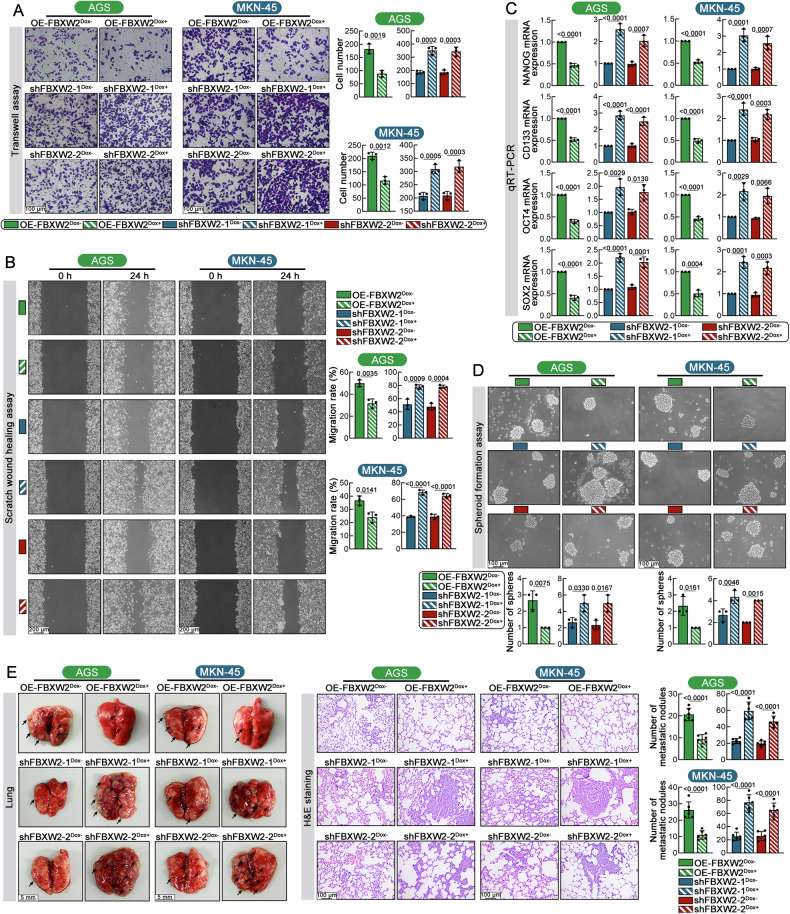
High FBXW2 expression curbs invasion, stemness and pulmonary metastasis of GC cells. **A** Transwell matrigel invasion assays were carried out to estimate the invasion ability of GC cells (Scale bars = 100 μm, magnification: ×200). **B** Scratch wound healing assays were carried out to estimate the migration ability of GC cells (Scale bars = 200 μm, magnification: ×100). **C** Real-time PCR was utilized to detect NANOG, CD133, OCT4 and SOX2 mRNA expression in GC cells. **D** Representative images and quantification of spheroid formation in GC cells (Scale bars = 100 μm, magnification: ×200). GC cells were injected into nude mice through the tail vein, and then the mice were fed 2 mg/ml doxycycline (Dox) in drinking water to induce FBXW2 expression. **E** Representative images of the whole lung tissues (Scale bars = 5 mm) and H&E staining (Scale bars = 100 μm, magnification: ×200) along with quantification of lung metastatic nodules. Arrows indicate lung metastasis nodules. Data were expressed as mean ± SD.

Following in vitro assays were conducted to evaluate the influence of FBXW2 in tumor metastasis. Transwell matrigel invasion assay revealed more cells crossed the membrane in the OE-FBXW2^Dox+^ groups than in the OE-FBXW2^Dox-^ group (Fig. 3A). Wound healing migration assay showed accelerated wound closure in OE-FBXW2^Dox+^ group relative to the OE-FBXW2^Dox-^ group (Fig. 3B). FBXW2 silencing induced by Dox enhanced the migratory and invasive capabilities of GC cells (Fig. 3A, B). Gastric cancer stem cells (GC-CSCs) are a key factor in GC metastasis and recurrence due to their self-renew and multi-differentiation properties [22]. Here, we detected the expression of CSC-related pluripotency transcription factors, such as SOX2, NANOG, OCT4 and CSC-specific surface marker CD133 in both AGS and MKN-45 cells. Reduction of these CSC markers in GC cells was observed after overexpression of FBXW2 induced by Dox (Fig. 3C). In addition, High FBXW2 expression induced by Dox significantly decreased tumor sphere formation ability (Fig. 3D). Consistently, knockdown of FBXW2 promoted spheroid formation and enhanced stemness marker expression. These data suggested that FBXW2 overexpression curbed migration, invasion and stemness of GC cells in vitro.

To assess FBXW2’s metastatic capacity in vivo, GC cells carrying lentiviral inducible FBXW2 expressing or shRNA constructs were administered via tail vein injection to nude mice. Results showed a significant reduction in lung metastatic nodules in the OE-FBXW2^Dox+^ group, whereas both shFBXW2-1^Dox+^ and shFBXW2-2^Dox+^ groups displayed elevated metastatic burden relative to respective controls (Fig. 3E).

### WASL is a potential FBXW2-binding partner in GC cells

**Fig. 4:**
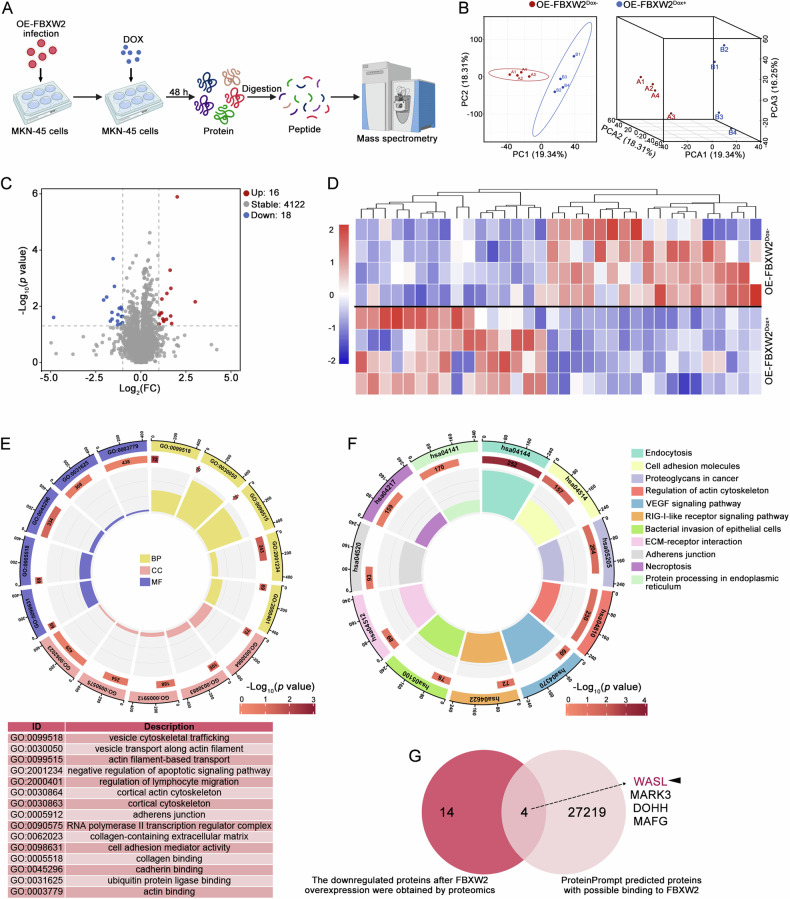
WASL is a potential FBXW2-binding partner in GC cells. **A** After treatment with doxycycline (Dox) for 48 h, FBXW2-overexpressed MKN-45 cells were subjected to label-free quantitative proteomics analysis. **B** 2D and 3D principal component analysis (PCA) plots. **C** The volcano plot of differential expressed proteins (DEPs; screening criteria: *p* < 0.05 and |log_2_FC|>1). **D** The heatmap of DEPs. **E** The circle plot of Gene ontology (GO) enrichment analysis of DEPs. **F** The circle plot of Kyoto Encyclopedia of Genes and Genomes (KEGG) enrichment analysis of DEPs. **G** By intersecting downregulated proteins identified by proteomics with FBXW2-binding proteins predicted by the ProteinPrompt, four overlapping proteins (WASL, MARK3, DOHH, MAFG) were obtained.

To identify the possible molecular mechanisms in which the ubiquitin ligase FBXW2 might be involved, we conducted a label-free quantitative proteomics on MKN-45 cells infected with lentiviral inducible FBXW2 expressing constructs under Dox (+/−) conditions, with four samples per group (Fig. 4A). The 2D-PCA and 3D-PCA plots displayed a clear separation between the OE-FBXW2Dox^+^ group and OE-FBXW2Dox^-^ group (Fig. 4B). A total of 34 DEPs were filtered by >2-fold change (|log₂FC|>1) and statistical significance (*p* < 0.05). Of the 34 DEPs, 16 were upregulated and 18 were downregulated (Fig. 4C). The results of GO and KEGG analyses revealed that FBXW2-mediated biological function in GC cells was involved in actin cytoskeleton reorganization and cell migration (Fig. 4E, F). By overlapping the downregulated DEPs with the potential FBXW2-binding proteins, four common proteins were identified. Among them, WASL had the highest binding score with FBXW2 and was expressed at the lowest level in FBXW2-overexpressed GC cells (Fig. 4G). Therefore, the potential FBXW2-binding partner WASL was used for follow-up mechanism exploration.

### FBXW2 induces WASL degradation via the ubiquitin-proteasome pathway

**Fig. 5:**
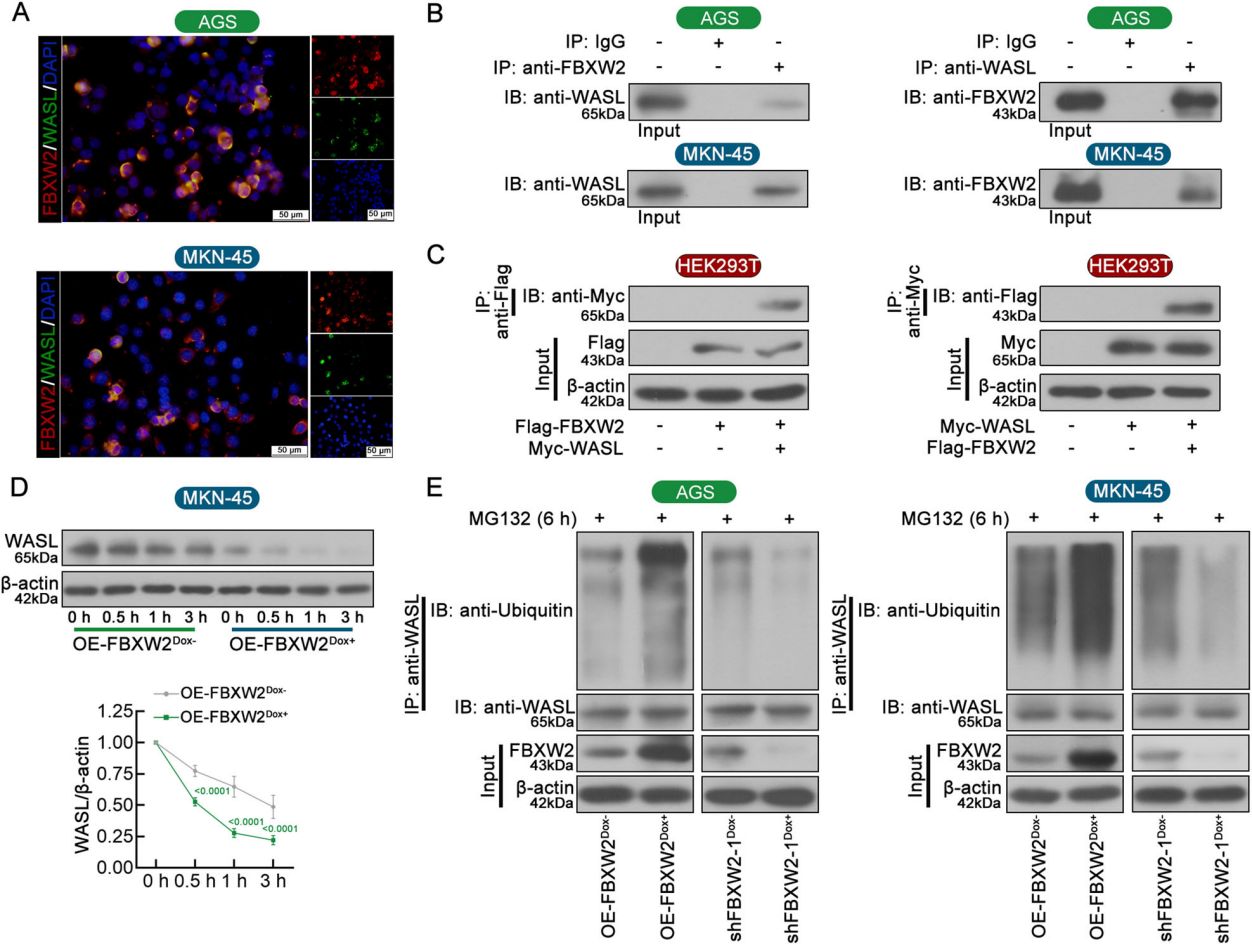
FBXW2 induces WASL degradation via the ubiquitin-proteasome pathway. **A** Colocalization of endogenous FBXW2 and WASL was visualized with double immunofluorescence staining (Scale bars = 50 μm, magnification: ×400). **B** Interaction between endogenous FBXW2 and WASL in GC cells was detected by co-immunoprecipitation (Co-IP). **C** Flag-tagged FBXW2 and Myc-tagged WASL were transfected into HEK293T cells. Co-IP and immunoblot (IB) analyses were carried out with the indicated antibodies. **D** After doxycycline (Dox) induction for 48 h, MKN-45 cells were treated with 100 µg/ml cycloheximide (CHX) for 0, 0.5, 1, and 3 h. Cell lysates were prepared for western blotting. **E** Co-IP assay was carried out to examine the ubiquitination levels of GC cells exposed, with or without Dox induction for 72 h, and exposed to MG132 (10 μM) for 6 h. IB was performed with anti-WASL and anti-ubiquitin. Data were expressed as mean ± SD.

To determine whether FBXW2 interacted with WASL in cells, we performed double immunofluorescence staining and Co-IP assay. Results revealed that FBXW2 interacted and co-localized with WASL in the cytoplasmic compartment of GC cells (Fig. 5A, B). The interaction between FBXW2 and WASL was subsequently confirmed by exogenous Co-IP analysis in HEK293T cells (Fig. 5C). After incubation with CHX to prevent further protein synthesis, FBXW2 overexpression induced by Dox led to faster degradation of WASL in GC cells (Fig. 5D). Furthermore, FBXW2 overexpression increased ubiquitination level of WASL in the presence of MG132 proteasome inhibitor, while FBXW2 silencing suppressed WASL ubiquitination (Fig. 5E). These results confirmed that FBXW2 induced WASL degradation via the ubiquitin-proteasome pathway.

Such degradation mediated by FBXW2 overexpression was blocked in the presence of MG132 proteasome inhibitor (Fig. 5E).

### WASL overexpression partially reverses the tumor-suppressing effects of FBXW2 in GC cells

**Fig. 6:**
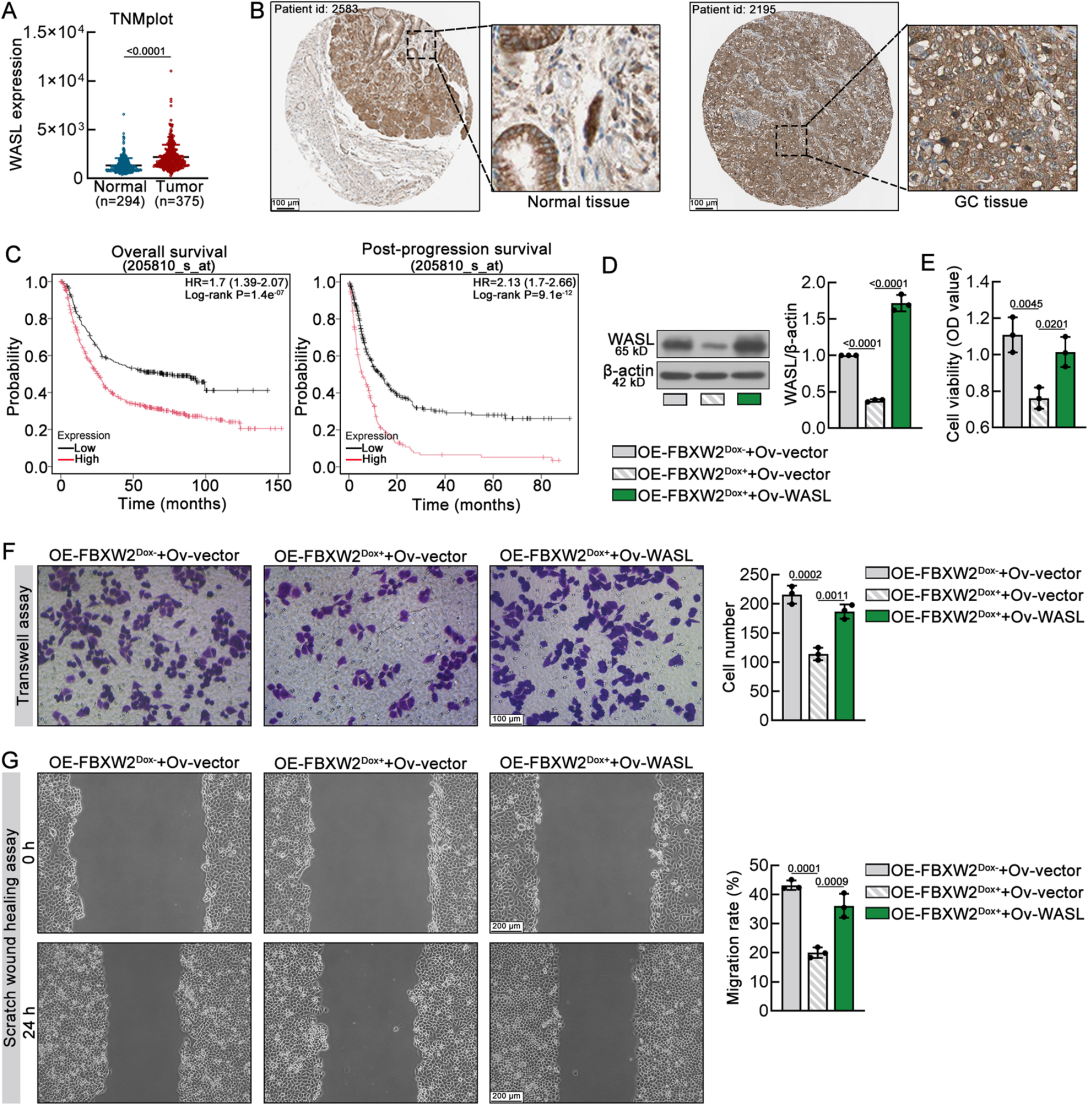
WASL overexpression partially reverses the tumor-suppressing effects of FBXW2 in GC cells. **A** Expression of WASL in the GC and normal tissues obtained from the TNMplot database. **B** Representative images of WASL immunohistochemistry in normal and GC tissues obtained from the Human Protein Atlas database. Scale bars = 100 μm. **C** Overall survival and post-progression survival analysis of WASL in GC patients obtained from the KM-plot database. **D** Representative western blotting and quantification of WASL in FBXW2-expressing MKN-45 cells. **E** The viability of MKN-45 cells was assessed using CCK-8 assays. **F** Representative images showing the invasion of MKN-45 cells (Scale bars = 100 μm, magnification: ×200). **G** Scratch wound healing assays were carried out to estimate the migration ability of MKN-45 cells (Scale bars = 200 μm, magnification: ×100). Data were expressed as mean ± SD.

Given that WASL was a binding partner of FBXW2, we investigated the role of WASL in GC. TNMplot, HPA and KM-plot databases suggested that WASL was an upregulated gene that predicts worse overall survival in GC patients (Fig. 6A–C). To determine whether WASL was required for FBXW2-mediated tumor-suppressing effects, we synthesized WASL-overexpressing plasmid to upregulation WASL expression (Supplementary Fig. S2). Then, we transfected the plasmid into MKN-45 cells stably expressing FBXW2. As shown in Fig. 6D, WASL overexpression inhibited the FBXW2-induced downregulation of WASL in MKN-45 cells. Functional experiments showed that overexpression of WASL partially reversed the inhibitory effects on cell proliferation, migration and invasion mediated by FBXW2 in GC cells (Fig. 6E–G).

### FOXP2 is responsible for low FBXW2 expression in GC cells

**Fig. 7:**
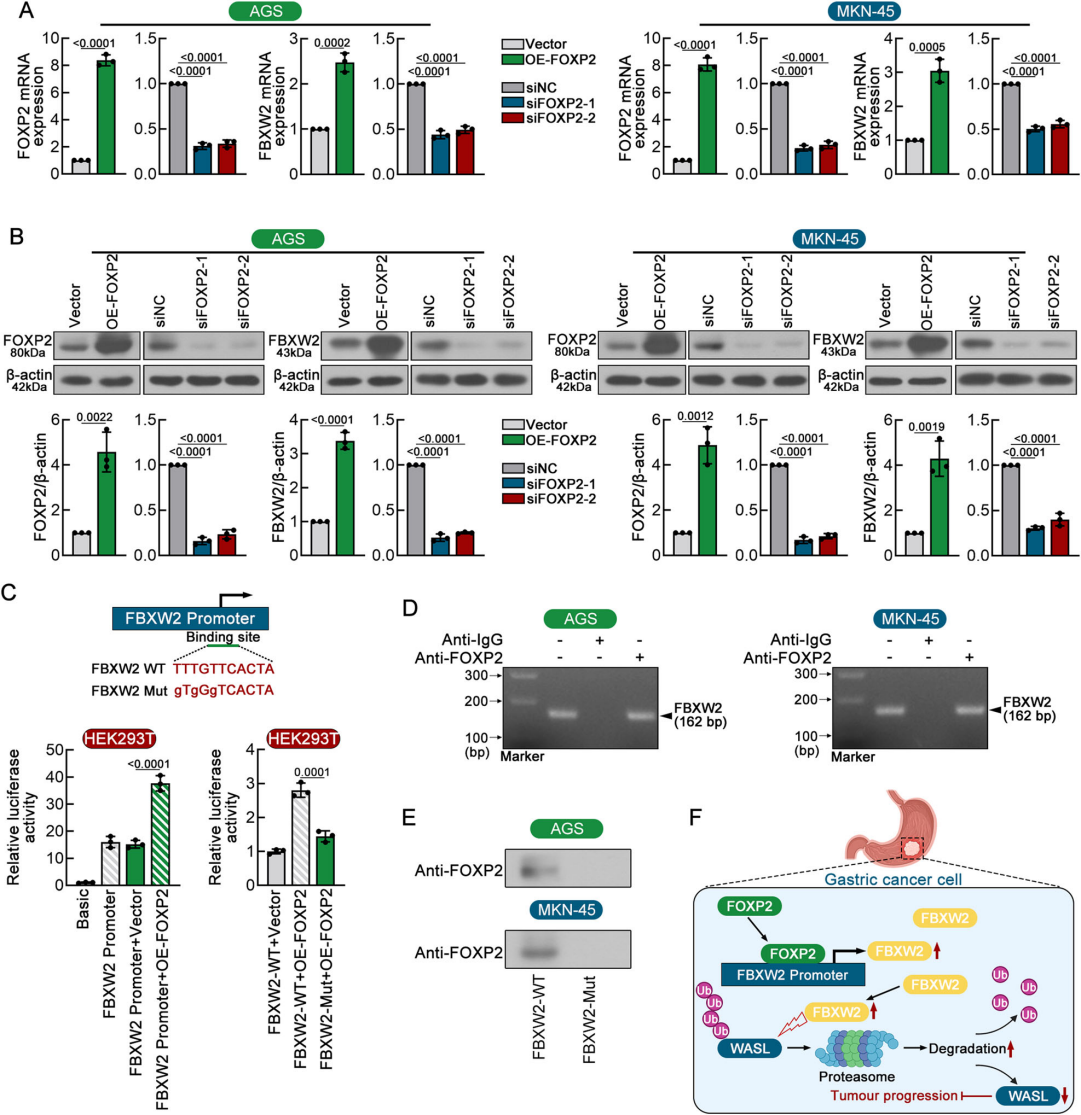
FOXP2 is responsible for low FBXW2 expression in GC cells. **A** Real-time PCR were used to detect the expression of FOXP2 and FBXW2 mRNA in GC cells. **B** Western blotting analysis. Top, representative protein bands. Bottom, relative optical density of FOXP2 and FBXW2 in GC cells was quantified via Gel-Pro-Analyzer software; β-actin was used as the internal control. **C** Dual-luciferase reporter assay was conducted with a wild-type (WT) FBXW2 and mutant (Mut) FBXW2 promoter conduct. The binding of FOXP2 to the FBXW2 promoter region was detected with the dual-luciferase reporter assay in HEK293T cells. **D** The binding site of FOXP2 protein to the FBXW2 promoter region was verified by chromatin immunoprecipitation (ChIP) assay in two GC cells. **E** DNA pull-down assay was used to verify this combine of FOXP2 and FBXW2 promoter. **F** Schematic illustration of the potential mechanism underlying FBXW2 tumor suppression in GC cells. Data were expressed as mean ± SD.

RNA-seq data from the TNMplot database demonstrated that FBXW2 mRNA expression was downregulated in gastric adenocarcinoma tissues compared with normal controls, prompting us to explore whether transcriptional regulation mechanisms modulated the levels of FBXW2 mRNA. The JASPAR database revealed a potential binding between FOXP2 and the promoter region of FBXW2. To investigate the regulatory role of FOXP2 on FBXW2 expression, FOXP2-interfering and -overexpressing plasmids were used to modulate FOXP2 expression in AGS and MKN-45 cells, followed by assessment of FBXW2 at both mRNA and protein levels (Fig. 7A, B). FBXW2 transcriptional and translational levels in GC cells was effectively reduced after transfection of FOXP2 siRNAs, yet enhanced after transfection of FOXP2 overexpression plasmid (Fig. 7A, B). Dual-luciferase reporter assay revealed that FOXP2 transcriptionally activated FBXW2 in HEK293T cells (Fig. 7C). Following ChIP-PCR assay was performed to confirm that FOXP2 bound to the FBXW2 promoter region covering the putative binding site in both AGS and MKN-45 cells (Fig. 7D). To further prove this result, dual-luciferase reporter assay was employed in combination with DNA pull-down assay to evaluate the functional consequences of FBXW2 mutations, using wild-type and T-to-G substituted FBXW2 constructs with single-nucleotide spacing. As demonstrated in Fig. 7C, high FOXP2 expression markedly enhanced the luciferase activity of the wild-type FBXW2 promoter, whereas the mutant promoter completely resisted the regulation mediated by FOXP2. DNA pull-down assay confirmed that mutant FBXW2 failed to bind to FOXP2 in both AGS and MKN-45 cells (Fig. 7E).

## Discussion

No signs or symptoms are exhibited in patients with early-stage GC. Most patients present with advanced-stage disease and thus lose opportunities for operation. The advanced GC patients have a median survival of ~10 months [2], therefore highlighting an urgent demand for novel tumor biomarkers and improved treatments. FBXW2 is considered a powerful therapeutic target for many other solid tumors, such as lung, breast, and prostate cancers, most of which display frequent KRAS mutations [5–8]. It is documented that overall median incidence of KRAS mutation in patients with GC is ~7% [23]. Using the KRAS mutant GC cell line (AGS) and the KRAS wild-type GC cell line (MKN-45), our data reveal that overexpression of FBXW2 exerts a tumor-suppressing role in both GC cell lines regardless of KRAS mutational status.

As the substrate recognizer, F-box proteins are critical to the function of SCF-type E3 ligases. F-box proteins are subdivided into three categories based on a recognizable domain, including (1) WD40 repeat-containing proteins (FBXW), (2) leucine-rich repeat-containing proteins (FBXL), and (3) other domain-containing proteins (FBXO) [24]. The FBXW subfamily contains 10 proteins, among which FBXW7 (also called hCDC4) is well-studied in GC [25, 26]. FBXW7, a well-characterized tumor suppressor, mainly functions by promoting the degradation of oncogenic regulators involved in cell cycle progression and cellular differentiation, such as cyclin E1, c-Myc, c-Jun and Notch [27]. Like FBXW7, FBXW2 exhibits an antioncogenic role in GC via inducing degradation of WASL. However, another FBXW protein, FBXW5, exerts a tumor-promoting effect in GC [28, 29]. These findings suggest that the FBXW proteins function either as tumor suppressors or promoters in GC, largely determined by the functions of the targeted substrates. A previous study has shown that β-TrCP1, one of the FBXW proteins (also called FBXW1), can trigger ubiquitination and degradation of FBXW2, thereby maintaining the stability of substrate protein and accelerating the growth of lung cancer cells. We note that β-TRCP1 is not expressed in any case of primary GC [30]. Therefore, β-TrCP1 may not act as an upstream regulator to mediate the biological role of FBXW2 in GC.

In the present work, FBXW2 expression in GC is downregulated after silencing of FOXP2, yet upregulated after overexpression of FOXP2. FOXP2 is generally regarded as a transcriptional repressor by binding to corepressors such as C-Terminal Binding Protein-1 (CtBP-1) and TLE Family Member 3 (TLE3) [31, 32]. However, it is also proven to activate some tumor suppressors, including PHD Finger Protein 2 (PHF2) and E-cadherin [17]. Xu et al. has been revealed that FOXP2 acts as a tumor suppressor in GC through mediating transcriptional inhibition of MET Proto-Oncogene (MET). It is necessary to understand why FOXP2 transcriptional inhibits FBXW2 in GC. In addition, what blocks the interaction between FOXP2 and corepressors demands further exploration in the future.

Invasion and metastasis are major malignant characteristics of GC that depend on actin cytoskeleton reassembly [33]. WASL is responsible for initiation of actin assembly via the ARP2/3 complex [10]. It has been documented that WASL-mediated invasive properties are induced by some signaling pathways triggered by estrogen, platelet-derived growth factor, and EGF [34–36]. In this work, FBXW2 overexpression leads to ubiquitination and degradation of WASL. This at least partly explains why the FBXW2 overexpression displayed low migratory and invasive capacities in vivo and in vitro. It is well-known that phosphorylation is prerequisite for the binding of a substrate to an F-box protein and subsequent ubiquitylation-guided degradation by the SCF complex [6]. FBXW2 usually recognizes phosphorylated serine/threonine residues on the consensus-binding (degron) motif (TSXXXS) [7]. Therefore, several issues merit further investigation in the future, including (1) how FBXW2 recruits the substrate WASL, (2) whether such recruitment hinges on degron phosphorylation, and (3) which kinase mediates degron phosphorylation and facilitates FBXW2-WASL binding.

Cancer stemness is essential for maintenance of malignant GC due to its renewal capacity. Gain- and loss-of-function experiments uncover that FBXW2 overexpression suppresses proliferation, stemness, migratory capacity, and invasive potential in human GC cells and tumor xenografts, while silencing of FBXW2 yield the opposite results. FBXW2 has been reported to reduce expression of several proteins associated with malignant tumor states, such as β-catenin [5], SKP2 [6], NF-κB p65 [8] and EGFR [7], which contribute to cancer stemness, tumorigenesis and metastasis [8, 37–39]. Yin et al. have claimed that overexpression of FBXW2 enhances expression of CSC marker SOX2 and promotes tumor sphere formation in breast cancer cells via inducing MSX2 degradation [9], which is contradictory with the abovementioned findings. More extensive studies are required to elucidate the contribution of FBXW2 in cancer stemness.

The current investigation used the tetracycline (Tet) inducible expression regulation system to control gene expression, where Dox serves as the preferrable inducer at optimized concentrations (~2.5 μg/mL) commonly used in such systems. The Tet-inducible system has been extensively utilized in cancer research, including GC studies, to elucidate the functional roles of specific genes [40–42]. Although Dox has reported anti-cancer effects at high concentrations [43, 44], our experimental conditions utilized concentrations well below cytotoxic thresholds. To address potential confounding effects, we included control groups expressing empty vector ± Dox in parallel experiments, which confirmed no significant phenotypic changes attributable to Dox alone (Fig. 2A, B). These findings were consistent with the previous research [40–42]. Data from this study shows that tumor growth rate and volume in the OE-FBXW2^DOX+^ group are significantly reduced relative to the OE-FBXW2^DOX−^ group. Furthermore, Dox-induced lentiviral gene interference system reveals that silencing of FBXW2 promotes tumor growth under Dox (+) condition. These data indicate FBXW2 overexpression-mediated tumor suppression rather than non-specific Dox effects.

Here, additional experiments are required to elucidate the prognostic significance of FBXWL2 in GC treatment. For instance, the expression levels of FBXWL2 in local clinical samples should be detected to mitigate potential biases arising from geographical disparities of publicly-available datasets. Alternatively, a clinicopathological correlation analysis is warranted to determine the impact of FBXWL2 expression on established clinical pathological parameters (such as tumor stage, histological differentiation and lymph node metastasis status). However, our current sample size is insufficient to conduct a robust statistical analysis. In addition to clinical validation, an orthotopic mouse model of GC can further assess the effect of FBXWL2 on native tumor microenvironment. Regrettably, the financial and time constraints prevented us from performing additional animal experiments at the present stage. Nevertheless, we are committed to addressing these limitations in future research endeavors to provide deeper insights into the roles of the FBXWL2 in GC.

In summary, this study identifies FBXW2 as an essential regulator of malignant GC by suppressing tumor stemness, tumorigenesis and metastasis, which may be at least partially attributed to its targeting of WASL. In addition, FOXP2 is responsible for FBXW2 loss in GC (Fig. 7F). FBXW2 may be a potential anti-metastasis target for GC treatment.

## Materials and methods

### TNMplot

TNMplot online analysis platform (www.tnmplot.com) was used for comparison of transcriptional profiles between tumor and normal tissue samples in gastric adenocarcinoma based on RNA sequencing (RNA-seq) datasets provided by The Cancer Genome Atlas.

### Human Protein Atlas (HPA)

Representative immunohistochemistry staining images of gastric adenocarcinoma specimens were acquired from the HPA public repository, accessible at https://www.proteinatlas.org/.

### Kaplan–Meier plotter (KM-plot) survival analysis

The KM-plot platform (https://kmplot.com/analysis/index.php?p=background), a comprehensive online tool for survival analysis, was applied to assess the clinical correlation between gene expression and survival outcomes, including both overall and post-progression survival in GC patients.

### Cell culture

Human GC cells AGS, KATOIII, Hs-746T, NCI-N87, HGC-27, MKN-45, and SNU-1, immortalized normal gastric epithelial cell GES-1, as well as HEK293T cells were supplied by Shanghai iCell Bioscience Inc. AGS cells were maintained in F12K medium (iCell-0007, iCell) supplemented with 10% fetal bovine serum (FBS). RPMI-1640 medium (31800, Solarbio, Beijing, China) containing 10% FBS sustained the growth of GES-1, NCI-N87, MKN-45, and SNU-1 cells. HGC-27, Hs-746T, and HEK293T cells were propagated in Dulbecco’s Modified Eagle Medium (G4510, Wuhan Servicebio Technology Co. Ltd., China) with 10% FBS, while KATOIII cells required IMDM (iCell-0008, iCell) with 20% FBS. All cells were maintained under standard conditions (37 °C, 5% CO₂, 95% humidity).

### Statistical analysis

Data underwent an analytical process carried out by GraphPad Prism 9.0.0. Continuous variables were subjected to normality testing via four complementary methods: D’Agostino & Pearson, Anderson-Darling, Shapiro–Wilk and Kolmogorov–Smirnov tests. Data passed normality testing were analyzed with unpaired two-tailed Student’s *t* test for two-group comparisons. One-way or two-way analysis of variance *post-hoc* Tukey’s or Sidak’s was applied for multiple-group comparisons. In all analyses, *p* < 0.05 was considered as statistical significance.

## Supplementary information

Supplementary information

original Western blot

## Data Availability

The datasets used during the current study are available from the corresponding author on reasonable request.
