# Supplementary material for: FOXP2 suppresses gastric cancer progression by transcriptionally repressing FBXW2 via WASL degradation
Source: Cell Death Discov. 2025 Jul 28;11:348. doi: 10.1038/s41420-025-02643-1 (PMC12304194; doi:10.1038/s41420-025-02643-1)
Supplement: Supplementary file 1 — Supplementary information [file 41420_2025_2643_MOESM1_ESM.docx]

**FOXP2 Suppresses Gastric Cancer Progression by Transcriptionally Repressing FBXW2 via WASL Degradation**

Sihan Lin^1^, Wencheng Kong^2^, Xinchun Liu^2^, Guang Yin^2^, Kangwen Cheng^2^, Zonglei Mao^2^, Yuqiang Shan^2*^, Xinger Lv^2*^

*^1^ Department of Emergency Surgery, Zhejiang Provincial People's Hospital, Hangzhou, Zhejiang, P.R.China*

*^2^* *Department of Gastroenterological Surgery, Affiliated Hangzhou First People's Hospital, School of Medicine, Westlake University, Hangzhou, Zhejiang, P.R.China*

^*^Correspondence: Xinger Lv (Email: Cmulxe@163.com, ORCID: 0000-0002-4146-9927, lead contact); Yuqiang Shan (Email: Shang110117@163.com, ORCID: 0000-0001-8397-6188)

**Supplementary materials and methods**

**Construction of lentiviral inducible plasmids**

pLVX-TetOne-Puro (FH1686, Fenghui Biotech, Hunan, China) and Tet-plko-Puro (BR250, Fenghui Biotech) lentiviral inducible vectors were used for overexpression and silencing of FBXW2.

For silencing of FBXW2, two FBXW2-specific short-hairpin RNAs (shRNAs) targeting sequences (5’-3’: GGAGAGAAAUCAACUGUAAGU and GAGUGUAUGCACUUUACUACA) or a non-specific control shRNA were inserted into the Tet-plko-Puro vector through AgeI/EcoRI restriction enzymes to construct Tet-plko-Puro-FBXW2 shRNA-1, Tet-plko-Puro-FBXW2 shRNA-2, and Tet-plko-Puro-NC shRNA. They were referred to simply as shFBXW2-1, shFBXW2-1, and shNC. For overexpression of FBXW2, the coding DNA sequence (CDS) fragment of FBXW2 (NCBI Accession: NM_012164) was inserted into between EcoRI and BamHI sites of pLVX-TetOne-Puro plasmid to construct pLVX-TetOne-Puro-FBXW2 expression plasmid, known simply as OE-FBXW2. An empty inducible expressing vector (EV) was regarded as control. The plasmids were transfected into HEK293T cells, where the corresponding lentiviruses were packaged.

AGS and MKN-45 cells were then infected with the above inducible lentiviruses, after which incubated in a medium containing puromycin to select stable clones. Doxycycline (Dox; 2.5 μg/ml) was applied to the clones for 48 h to induce overexpression and silencing of FBXW2. Infection efficiency in cells was detected by Real-Time PCR.

**RNA interference and plasmid transfection**

To determine whether FOXP2 was responsible for low FBXW2 expression within GC, we constructed two FOXP2-specific small interfering RNAs (siRNAs) and an FOXP2 expression plasmid. Two siRNAs targeting CDS and 3’untranslated region (3’UTR) of FOXP2 mRNA were designed (5’-CGACAGAGACAAUAAGCAA-3’ for siFOXP2-1, and 5’-GAGUUAUGAUGUAGUUGAA-3’ for siFOXP2-2). A negative control (siNC) was established using a non-targeting siRNA. For FOXP2 overexpression, the FOXP2 CDS fragment (NCBI Accession: NM_014491) was inserted into the pCDNA3.1 plasmid. The plasmid or siRNAs was then transfected into AGS and MKN-45 cells. Following 48 h, the cells were subjected to Dox treatment for an additional 48 h. Transfection efficiency in cells was detected by Real-Time PCR and western blotting assays.

To detect the binding between exogenous FBXW2 and WASL, HEK293T cells were co-transfected with a FBXW2 pcDNA3.1 plasmid tagged at the C-terminus with Flag and a WASL pcDNA3.1 plasmid tagged at the C-terminus with Myc, using lipofectamine 3000 as per the manufacturer’s instructions.

**Cell viability assay via cell counting kit-8 (CCK-8)**

A density of 7 × 10^3^ GC cells was inoculated into 96-well plates and subjected to 2.5 μg/ml of Dox at dictated time. CCK-8 reagent (10 μl; KGA317, KeyGen, Nanjing, China) was pipetted into each well, followed by a 2-h incubation at 37°C to facilitate the reaction between the CCK-8 solution and the living cells. Once the reaction was complete, the absorbance values at 450 nm were measured to quantify the cell viability.

**Colony formation assay**

Cell proliferation ability was assessed via colony formation assay using a Wright-Giemsa Stain Kit (D011-1-2, Jiancheng Bioengineering Institute, Nanjing, China). Single-cell suspensions (300 cells) were inoculated into a culture dish containing complete medium supplemented with containing 2.5 μg/ml of Dox. After two weeks, the cultures were immobilized using R1 regent for 60 s and subjected to coloration with R2 regent for 5 min. The colony formation efficiency was determined as the ratio of the number of colonies formed relative to the initial number of inoculated cells.

**Transwell matrigel invasion assay**

Cell invasion capacity was measured using a two-chamber system separated by an 8μm pore-size membrane insert (Corning Costar, Cambridge, MA, USA). A 200 μl suspension of 5 × 10^4^ cells in serum-free medium containing Dox (2.5 μg/ml) were added to the matrigel-coated upper chamber of a Transwell system. The lower chambers were filled with 800 μl of complete growth medium supplemented with 10% FBS to induce cell migration. Following 24 h of culture, non-invading cells were gently removed from the upper membrane surface using sterile cotton-tipped applicators. The inserts were then subjected to fixation in 4% paraformaldehyde (20 min) and immersed in a 0.5% crystal violet staining (5 min). Finally, images of invading cells adhering to the lower surfaces of inserts were captured via an OLYMPUS IX53 inverted microscope at 200 × magnification, and cell counts were quantified using Image J software.

**Wound healing migration assay**

Cells were maintained as a monolayer to 90% confluence for wound healing assay. Scratch wounds were generated via a disposable 200-μl pipette tip in the presence of mitomycin C (1 μg/ml). Wound closure was imaged at 0 and 24 h post-serum starvation using the OLYMPUS IX53 inverted microscope at 100 × magnification. Cell migration rates were determined via the formula: (T_0_-T_24_)/T_0_, where T_0_ is wound width at baseline after creating the wound and T_24_ is wound width after 24 h.

**Spheroid formation assay**

GC cells were cultured in serum-deprived medium containing 2% B-27 (BL319A, Biosharp Biotech, Hefei, China), 20 ng/ml EGF (HY-P7109, MedChemExpres, Monmouth Junction, NJ, USA) and 10 ng/ml bFGF (HY-P70600, MedChemExpres). These cells were seeded at a density of 500 cells per well in 12-well ultra-low-attachment plates and cultured under standard conditions (37°C, 5% CO_2_, humidified atmosphere). After seven days, the cultures were examined for tumor sphere formation. Representative images were acquired via the OLYMPUS IX53 inverted microscope at 200 × magnification.

***In vivo* animal studies**

All experimental procedures involving animals were conducted in strict accordance with the National Institutes of Health Guidelines for the Care and Use of Laboratory Animals, following protocol approval from the institutional animal care and use committee at animal research center of Zhejiang Provincial People’s Hospital. Eight-week-old male BALB/c nude mice were maintained in standard conditions (temperature of 22 ± 1℃, humidity of 45-55% and 12-h photoperiod). Mice were provided with free access to food and water throughout the study. Following a 7-day acclimatization period, the mice were subcutaneously injected with 200 μl of GC cells carrying lentiviral constructs (3 × 10^6^ MKN-45 cells or 1 × 10^7^ AGS cells). When the tumor was visible to the naked eye, the mice were randomized into two groups (6 mice/group). The mice in the Dox^+^ group received drinking water containing 2 mg/ml Dox dissolved in 5% glucose, while the mice in the Dox^-^ group received 5% glucose water alone. Tumor volume was measured every three days using a caliper via the measurement equation: D × d^2^ × 0.5, with D representing the major diameter and d the minor diameter. Following a 30-day observation period, the mice were euthanized. Tumors were harvested, photographed, weighed and used for immunohistochemistry assay. For *in vivo* pulmonary metastasis, tail vein injection of 2 × 10^6^ cells was performed in nude mice. Mice were available drinking water containing 5% sucrose with and without Dox (2 mg/ml). After four weeks, the mice were euthanized, after which pulmonary tissues were removed, photographed, and used for hematoxylin&eosin (H&E) staining. The maximal tumor size was not exceeded 17 mm.

**Histopathological evaluation**

For immunohistochemistry assay, the tissues underwent fixation in 4% paraformaldehyde, followed by paraffin-embedding and sectioning at 5-μm thickness. Following deparaffinization in xylene, rehydration with graded ethanol and subsequent heat-antigen retrieval, the tissue sections were exposed to 3% H_2_O_2_ (15 min) at room temperature (RT) to eliminate endogenous peroxidase activity. Non-specific binding sites were blocked via 15-min incubation with 1% bovine serum albumin (BSA). Afterward, the slides were treated with the primary antibodies against FBXW2 (11499-1-AP, Proteintech Group Inc., Rosemont, IL, USA [1:200 dilution]) and Ki67 (28074-1-AP, ProteinTech [1:200 dilution]) at 4°C overnight. Horseradish peroxidase (HRP)-labeled secondary antibody (goat anti-rabbit IgG; SE134, Solarbio [1:100 dilution]) was applied for 45 min at RT to detect primary antibody binding. Following development with 3,3’-diaminobenzidine (DAB) chromogen solution, the slides were counterstained using hematoxylin and mounted with neutral balsam. Immunohistochemistry staining was visualized via an OLYMPUS BX53 microscope at 400 × magnification.

H&E staining was performed by inducing deparaffinized and rehydrated sections with hematoxylin for 5 min, followed by eosin-counterstaining for 3 min. After dehydration in graded ethanol and xylene, sections were mounted with neutral balsam. H&E staining was observed via the OLYMPUS BX53 microscope at 200 × magnification.

**Label-free quantitative proteomics**

MKN-45 cells were infected with lentiviral inducible FBXW2 expressing constructs under Dox (+/-) conditions (four samples per group). Total proteins from the abovementioned cells were extracted with lysis buffer supplemented with protease inhibitors and resolved via SDS-PAGE. Qinglianbio Biotech (Beijing, China) conducted qualitative proteomic analysis of precipitated proteins using a RIGOL L-3000 HPLC System for protein separation coupled with Proteome Discoverer 2.4 for comprehensive data analysis. The protocol was shown in **Fig. 4A**.

**Bioinformatics analyses**

The dataset was subjected to principal component analysis (PCA) utilizing R (v4.3.2) with ggplot2 (v3.5.0) and scatterplot3d (v0.3.44) to generate two-dimensional (2D) and three-dimensional (3D) projections. Volcano plot and heatmap of differentially expressed proteins (DEPs) were drawn using R (v4.3.2) ggplot2 (v3.5.0) and pheatmap (v1.0.12) with significance thresholds set at p-value < 0.05 and |log_2_FlodChange (FC)| > 1. R (v4.3.2) was used to execute GO (BP/MF/CC) and KEGG pathway enrichment analysis of DEPs via the clusterProfiler (v4.10.1) and human genome annotation database (org.Hs.eg.db, v3.18.0). To identify potential FBXW2-binding proteins, ProteinPrompt online server (https://proteinformatics.uni-leipzig.de/ProteinPrompt/) was utilized for sequence-based interaction prediction. Venn analysis was applied to intersect DEPs from proteomics data with FBXW2-interacting proteins, prioritizing downregulated candidates for further validation.

**Double immunofluorescence staining**

Following fixation with 4% paraformaldehyde, cells were permeabilized using 0.1% Triton X-100 prior to 1% BSA blocking and primary antibody incubation at 4°C overnight. The primary antibodies used were as stated below: anti-FBXW2 antibody (11499-1-AP, ProteinTech [1:100 dilution]) and anti-WASL antibody (sc-271484, Santa cruz Biotech, Shanghai, China [1:100 dilution]). After triple PBS washes, the cells underwent a 60-min incubation at RT under light-protected conditions with goat-derived Alexa Fluor® 555-coupled anti-rabbit IgG (4413, CST, Danvers, MA, USA) or goat-derived Alexa Fluor® 488-coupled anti-mouse IgG (4408, CST) secondary antibody, both diluted at a ratio of 1:200. Counterstaining of nuclei was performed with 4’,6-diamidino-2-phenylindole (DAPI). Finally, the slides were mounted with an anti-fluorescent quencher. Representative images were captured by the OLYMPUS BX53 microscope at 400 × magnification.

**Co-immunoprecipitation** **(Co-IP) and western blotting assays**

Whole cell lysates were prepared from both GC and HEK293T cells using RIPA lysis (PR20001, ProteinTech) containing protease inhibitor cocktail (PR20032, ProteinTech). The cell lysates were immunoprecipitated using AminoLink™ Plus Coupling Resin (26149, ThermoFisher, Pittsburgh, PA, USA) conjugated with IP-graded antibody against FBXW2 (11499-1-AP, ProteinTech), Flag (66008-4-Ig, ProteinTech), Myc (16286-1-AP, ProteinTech) or WASL (sc-271484, Santa cruz Biotech). The immunoprecipitated complexes were eluted and used for western blotting analysis.

For western blotting assay, protein samples (15-30 μg) were electrophoresed on 12% SDS-polyacrylamide gels, after which electroblotted onto polyvinylidene difluoride membranes. Following treatment with 5% non-fat dry milk, the membranes were subjected to overnight incubation with primary antibody at 4°C, followed by a 40-min incubation at 37°C with goat-derived HRP-conjugated anti-rabbit (SA00001-2, ProteinTech) or goat-derived HRP-conjugated anti-mouse IgG (SA00001-1, ProteinTech) secondary antibody, both diluted at a ratio of 1:10000. Proteins were visualized with an Enhance Chemiluminescence Reagent (PK10003, ProteinTech). The following primary antibodies used in this assay were applied at indicated dilutions: anti-FBXW2 antibody (11499-1-AP, ProteinTech [1:500 dilution]), anti-WASL antibody (sc-271484, Santa cruz Biotech, [1:500 dilution]), anti-Flag antibody (66008-4-Ig, ProteinTech [1:5000 dilution]), anti-Myc antibody (16286-1-AP, ProteinTech [1:1000 dilution]), anti-Ubiquitin antibody (10201-2-AP, ProteinTech [1:500 dilution]) and anti-FOXP2 antibody (20529-1-AP, ProteinTech [1:500 dilution]). Full and uncropped western blot bands were shown in Supplemental Material.

**DNA pull down**

Biotinylated DNA probes targeting FBXW2 promoter were synthesized and coupled with streptavidin magnetic beads. Nuclear lysates isolated from AGS and MKN-45 cells were reacted with probe-conjugated beads under cold conditions (4°C) for 60 min. Following purification and elution, the mixtures were subjected to western blotting using anti-FOXP2 antibody.

**Protein stability and degradation experiment**

MKN-45 cells stably transfected with lentiviral inducible FBXW2 expressing constructs were treated with Dox (2.5 μg/ml) for 48 h to induce FBXW2 overexpression. For cycloheximide (CHX) chase experiments, cells were incubated with CHX (10 μg/ml) for indicated times to block de novo protein synthesis. Total protein extracts were harvested at each time point and subjected to western blotting analysis.

To assess whether FBXW2-mediated WASL degradation was associated ubiquitin-proteasome pathway, MG132 (10 μM), a proteasome inhibitor, was introduced into cell culture medium to block protein degradation. After 6 h of MG132 treatment, total protein was extracted for western blotting.

**Real-Time PCR**

TRI Reagent (RP1001, Bioteke Biotech, Beijing, China) was utilized to extract total RNA from GC cells. First-strand cDNA synthesis was conducted using an All-in-One SuperMix Kit (Magen Biotech, Guangzhou, China) with 1 μg of RNA. The obtained cDNA (1 μl) was then used for Real-Time PCR reaction with SYBR Green I nucleic acid stain (0.3 µl; SY1020, Solarbio), 2 × DNA polymerase master mix (10 µl; PC1150, Solarbio), and each primer (1 μl). The primer sequences utilized in this assay were shown in Table S1. Gene expression levels were quantified by the 2^-△△CT^ method and normalized in accordance with the expression level of β-actin.

**Dual-luciferase reporter assay**

Potential binding between FOXP2 and promoter region of FBXW2 was revealed by JASPAR online website (https://jaspar.elixir.no/). Dual-luciferase reporter assay was used to assess whether FBXW2 was transcriptionally regulated by FOXP2. HEK293T cells cultivated in 12-well plates were co-transfected with the following plasmids: FOXP2-expressing pCDNA3.1 plasmid or empty plasmid (1 μg), pGL3-Basic carrying FBXW2 promoter fragment (-1720~+100) or empty reporter plasmid (1 μg), and pRL-TK plasmid (1 μg). For promoter mutagenesis, the wild-type FOXP2 binding motif (5’-TTTGTTCACTA-3’) was mutated to 5’-gTgGgTCACTA-3’, where the thymine (T) residues at positions 1, 3, and 5 were replaced by guanine (G). Forty-eight h after transfection, relative luciferase activity was determined using the Dual-Luciferase Reporter Assay System (KGAF040, KeyGen) that was adjusted based on Renilla luciferase activity.

**ChIP assay**

ChIP experiments were conducted by means of a ChIP Assay Kit (P2078, Beyotime, Shanghai, China) in strict accordance with the supplier’s instructions. Cells were fixed in 1% formaldehyde, lysed and sonicated into DNA fragments with several hundreds of base pairs. Chromatin was subjected to immunoprecipitation using FOXP2 antibody or nonspecific IgG at 4°C throughout the night, followed by binding to protein G agarose for 60 min under same temperature (4°C). Immunoprecipitated DNA was eluted and detected by Real-Time PCR. The specific primers targeting FBXW2 promoter region were designed based on a putative FOXP2-binding site with JASPAR score > 8. The primer sequences were as shown in Table S2.

**Supplementary figure and tables**


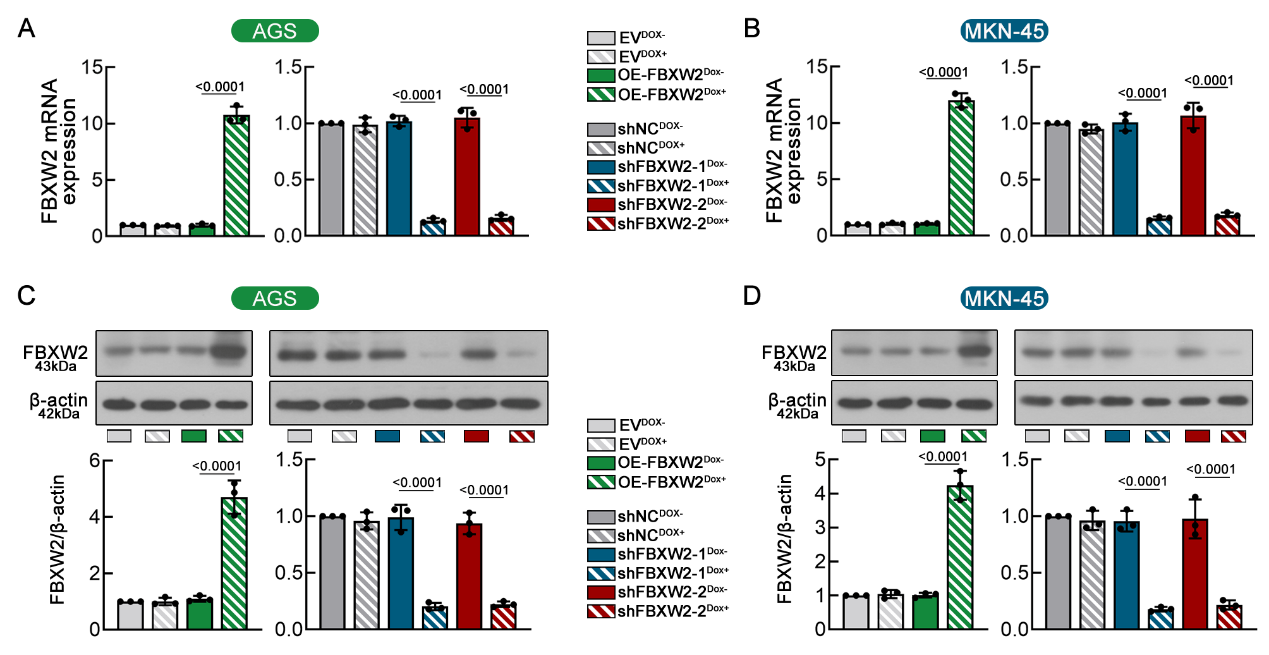


**Fig. S1 The efficiency of FBXW2 knockdown and overexpression, related to Fig. 2**

(A, B) Real-time PCR for the FBXW2 mRNA in GC cells with or without doxycycline (Dox) induction. (C, D) Western blotting analysis. Top, representative protein bands. Bottom, relative optical density of FBXW2 in GC cells with or without Dox induction. was quantified via Gel-Pro-Analyzer software; β-actin was used as the internal control. Data were expressed as mean ± SD.


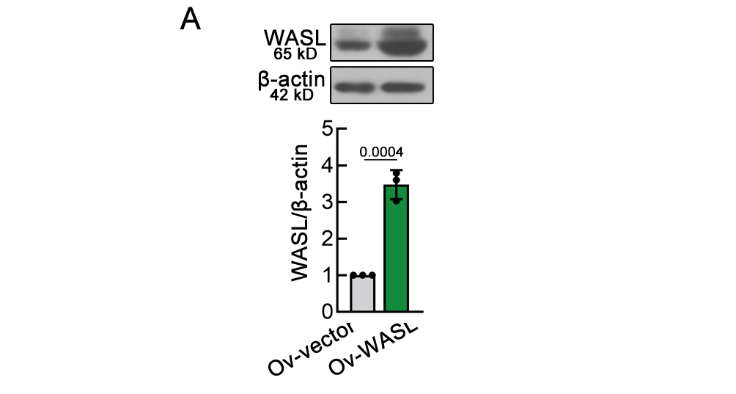


**Fig. S2 The efficiency of WASL overexpression, related to Fig. 6**

(A) Western blotting analysis. Top, representative protein bands. Bottom, relative optical density of WASL in MKN-45 cells was quantified via Gel-Pro-Analyzer software; β-actin was used as the internal control. Data were expressed as mean ± SD.

**Table S1. The primer sequences used in Real-Time PCR.**

| **Gene** | **Sequence (5’-3’)** |
| --- | --- |
| CD133 | Forward: CCAAGGACAAGGCGTTCA |
|  | Reverse: GCACCAAGCACAGAGGG |
| FBXW2 | Forward: CTTTGCCCTGCTGTTTG |
|  | Reverse: AAGCCCGTGTCATTGTG |
| FOXP2 | Forward: GGGGCCTCTCACACTCTCTA |
|  | Reverse: CACCACCTGCATTTGCACTC |
| OCT4 | Forward: AGGGCAAGCGATCAAGC |
|  | Reverse: GGAAAGGGACCGAGGAGTA |
| SOX2 | Forward: ATGCACCGCTACGACGTGAG |
|  | Reverse: GCCCTGGAGTGGGAGGAAGA |
| NANOG | Forward: CTGCAGAGAAGAGTGTCGCA-3 |
|  | Reverse: ATCTGCTGGAGGCTGAGGTA |
| β-actin | Forward: GGCACCCAGCACAATGAA |
|  | Reverse: TAGAAGCATTTGCGGTGG |

**Table S2. The primer sequences used in ChIP assays.**

| **Gene** | **Sequence (5’-3’)** |
| --- | --- |
| FBXW2 | Forward: ACCTCCTTACCTTGCTTTATT |
|  | Reverse: CTGTTGGGCACCTCTTTA |
