# Supplementary material for: FOXP2 suppresses gastric cancer progression by transcriptionally repressing FBXW2 via WASL degradation
Source: Cell Death Discov. 2025 Jul 28;11:348. doi: 10.1038/s41420-025-02643-1 (PMC12304194; doi:10.1038/s41420-025-02643-1)
Supplement: Supplementary file 2 — original Western blot [file 41420_2025_2643_MOESM2_ESM.pdf]

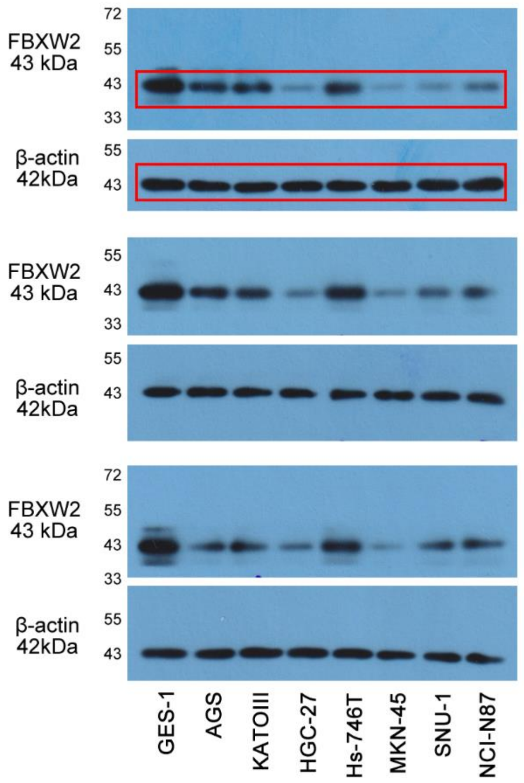

WBoriginal-Fig1

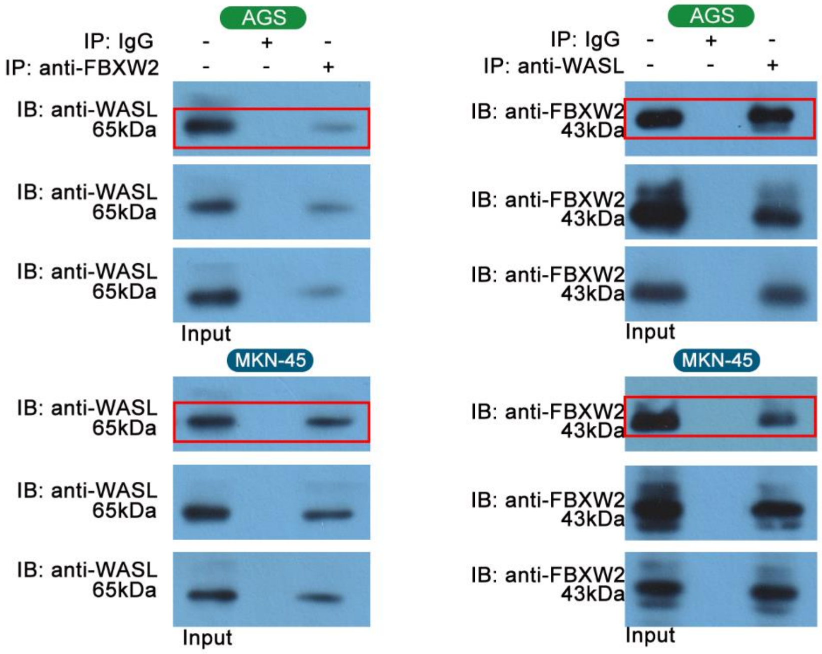

WBoriginal-Fig5B

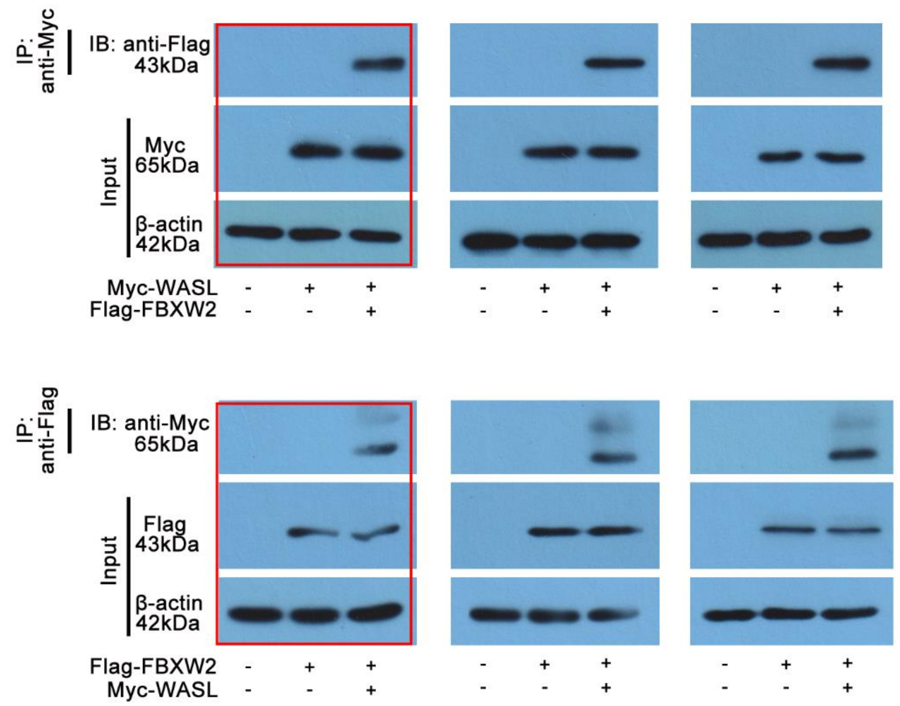

WBoriginal-Fig5C

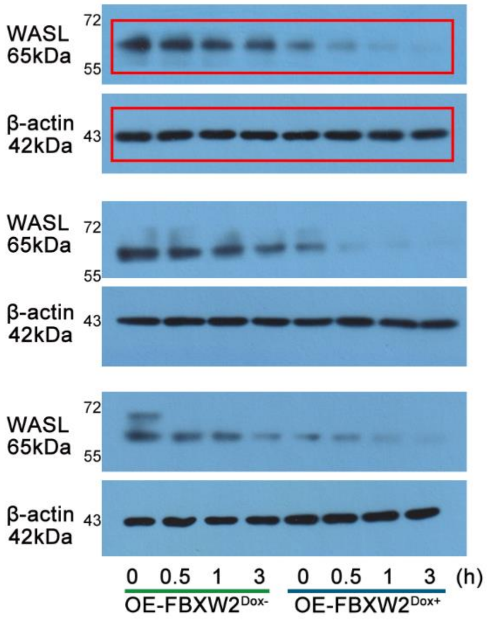

WBoriginal-Fig5D

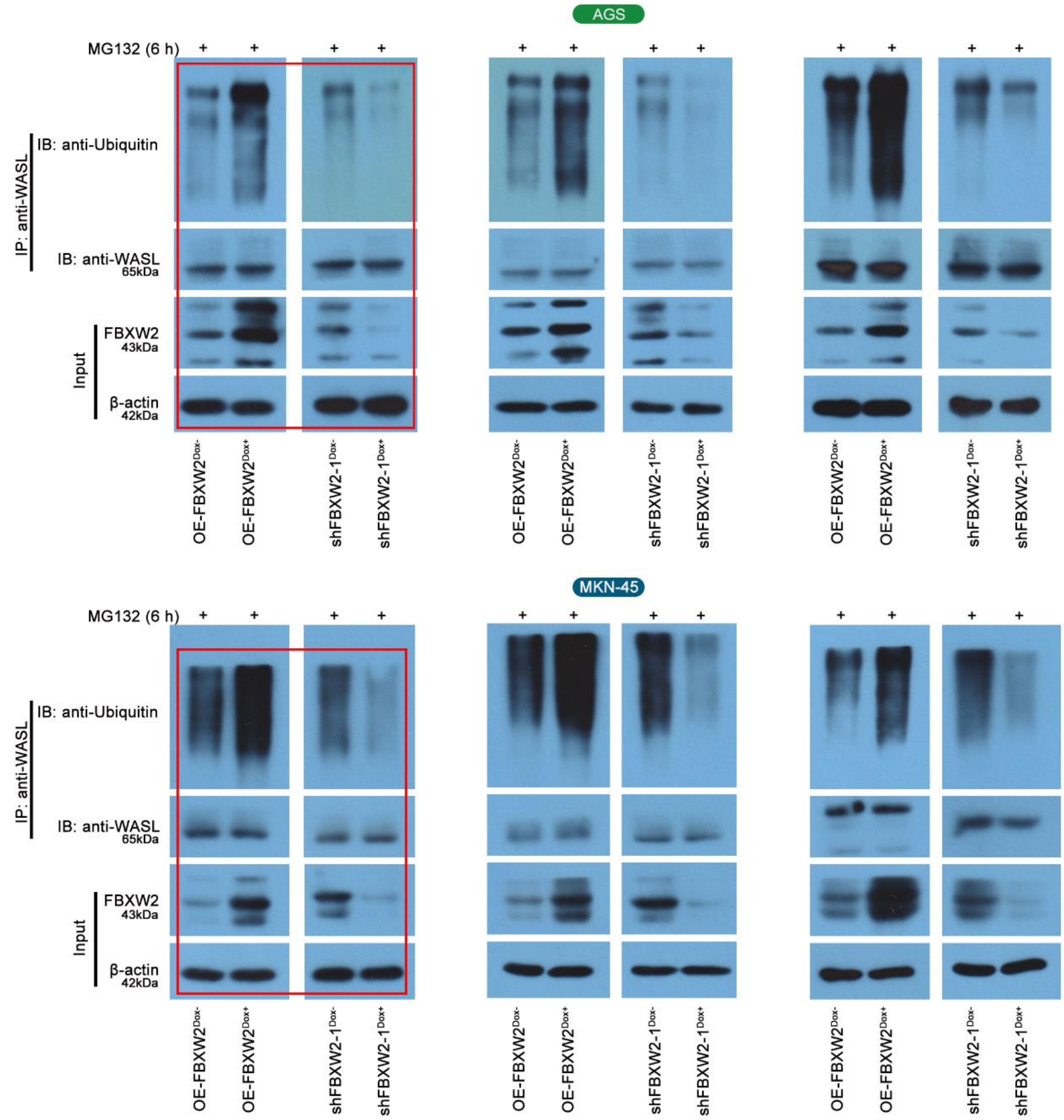

WBoriginal-Fig5E

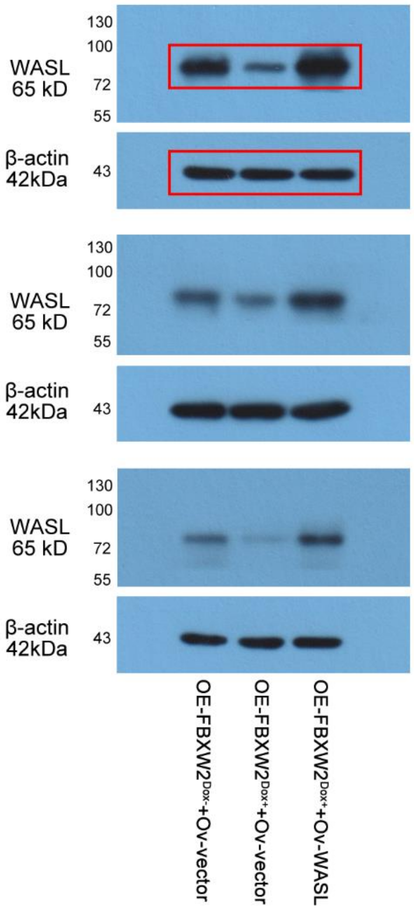

WBoriginal-Fig6D

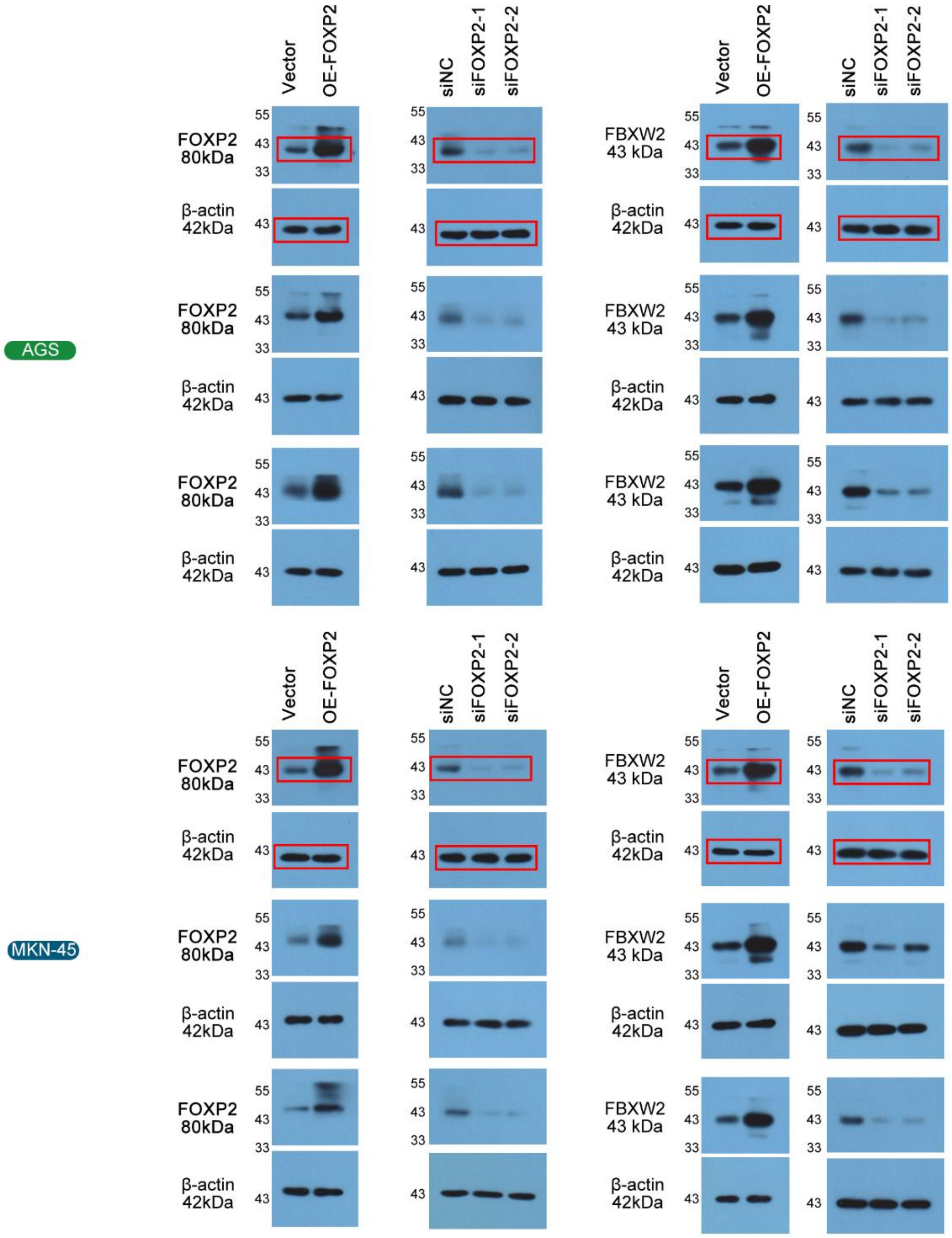

WBoriginal-Fig7B

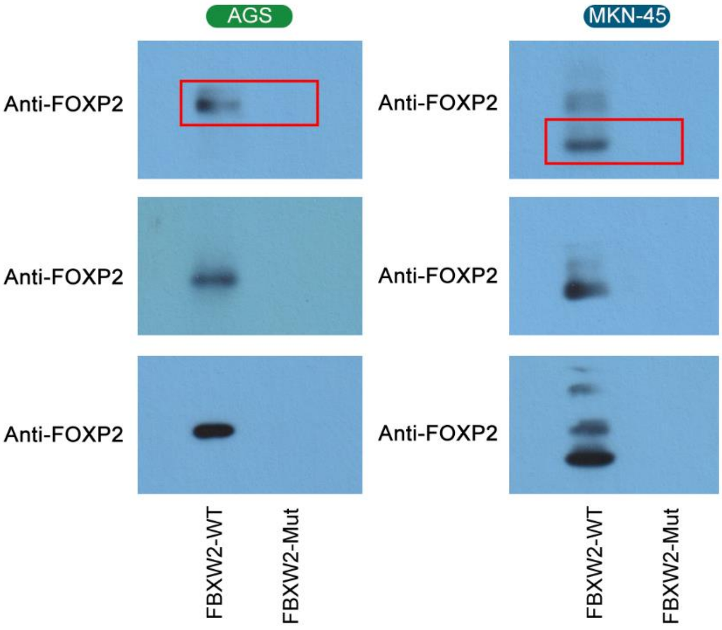

WBoriginal-Fig7E

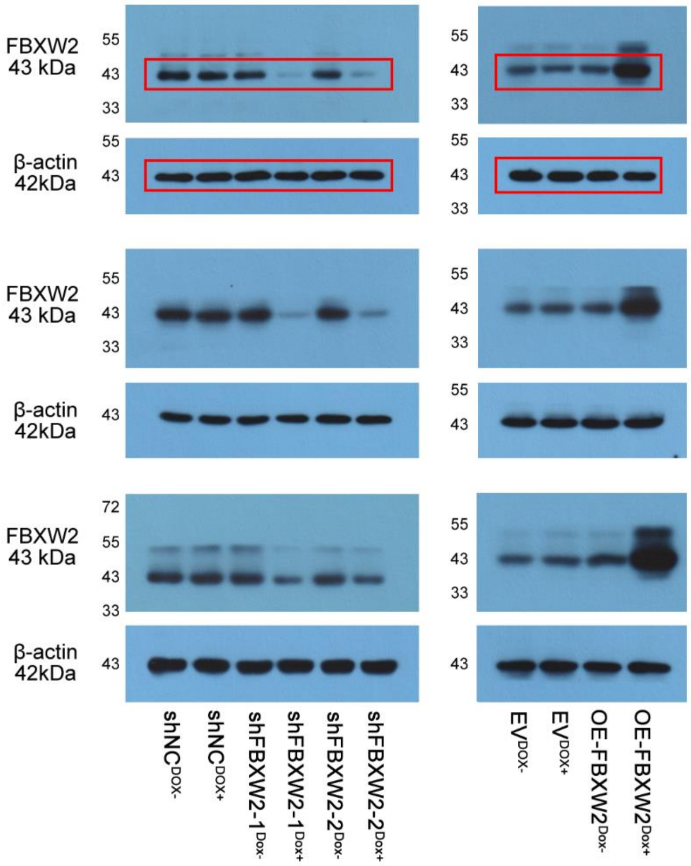

WBoriginal-FigS1C

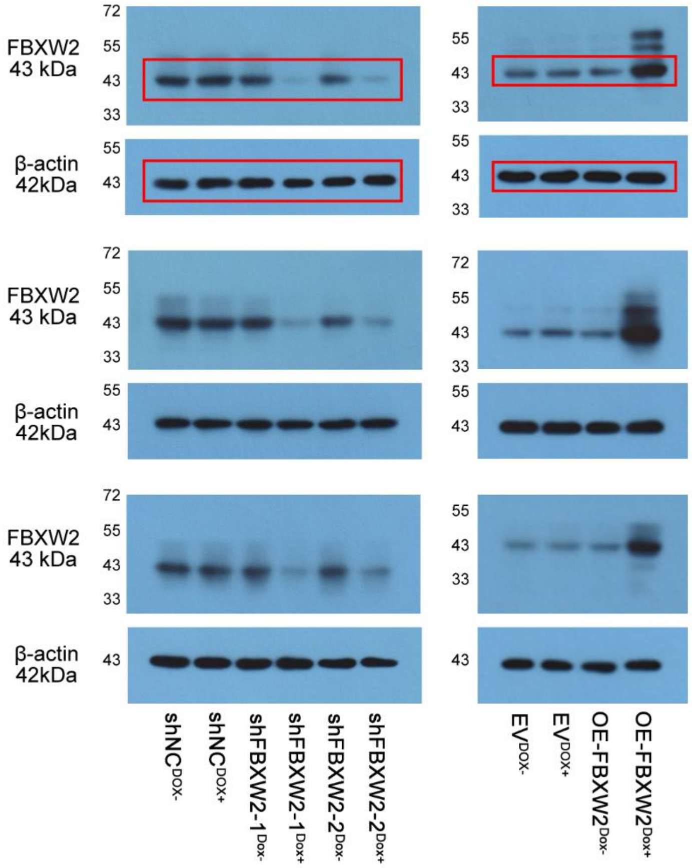

WBoriginal-FigS1D

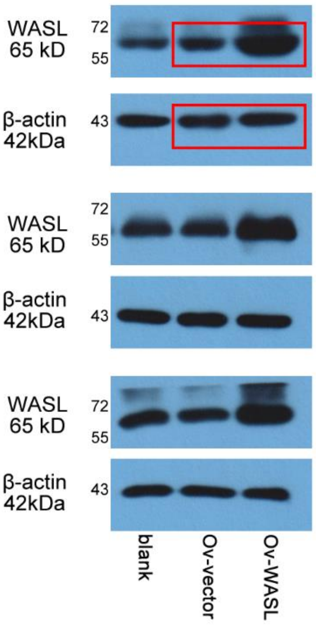

WBoriginal-FigS2A
